# Thymoquinone and Its Nanoformulation for Dual Antiviral Therapy: Targeting Intracellular Viral Replication and Host Complications

**DOI:** 10.3390/biom16101408

**Published:** 2026-09-28

**Authors:** Masood Alam Khan, Mohammad Hamza Khan, Khaled S. Allemailem

**Affiliations:** 1 Department of Basic Health Sciences, College of Applied Medical Sciences, Qassim University, Buraydah 51452, Saudi Arabia; 2 Jawaharlal Nehru Medical College and Hospital (JNMCH), Aligarh Muslim University, Aligarh 202002, India; 3 Department of Medical Laboratories, College of Applied Medical Sciences, Qassim University, Buraydah 51452, Saudi Arabia; k.allemailem@qu.edu.sa

**Keywords:** thymoquinone, nanoparticles, antiviral activity, inflammation, COVID-19

## Abstract

Viral therapy requires effective control of intracellular viral replication while limiting harmful host responses that may contribute to disease progression and complications. Thymoquinone (TQ), a major bioactive constituent of *Nigella sativa*, has potential antiviral and host-protective properties, but its therapeutic implications are limited by poor aqueous solubility, chemical instability, and variable tissue exposure. This review critically explores TQ nanoformulations (Nano-TQ) as a promising strategy to overcome these limitations and enable dual antiviral therapy through improved intracellular delivery and modulation of virus-associated host injury. Research published between 2000 and 2026 is evaluated, with emphasis on TQ-specific antiviral activity, nanoformulation platforms, intracellular trafficking, virus-informed delivery, host complications, and translational development. Current evidence indicates that nanoformulation can improve the pharmaceutical and pharmacokinetic properties of TQ and may facilitate delivery to disease-relevant tissues. However, direct evidence that Nano-TQ can achieve functionally relevant intracellular delivery, selectively engage virus-related targets, or provide superior antiviral efficacy over free TQ remains limited. We therefore propose that the key translational benchmark should be a reproducible therapeutic advantage coupled to intracellular TQ exposure, target engagement, and reduced infectious viral output rather than improved bioavailability alone. Future studies should integrate virus-specific infection models, PK–PD relationships, and safety assessments to progress Nano-TQ toward a clinically relevant dual-action antiviral nanomedicine.

## 1. Introduction

Viral infections remain a major global health challenge despite substantial advances in vaccines and antiviral therapy [1]. Acute respiratory infections can advance promptly to severe disease, whereas chronic viral infections may establish persistent intracellular pools that are difficult to eliminate [2]. The emergence of new strains of viruses and antiviral resistance further emphasizes the need for therapeutic approaches that extend beyond conventional virus-specific approaches. Because viruses replicate intracellularly, effective antiviral molecules must reach infected tissues and achieve therapeutically relevant concentrations. The clinical consequences of viral infections are not determined solely by viral replication. But dysregulated host responses can also contribute substantially to disease severity through excessive inflammation, oxidative stress, endothelial injury, and immune-cell dysregulation [3]. These viral diseases may therefore be complicated by acute lung injury, acute respiratory distress syndrome, cardiovascular injury, coagulopathy, multiorgan dysfunction, and secondary microbial infections [4]. This was particularly evident during severe Coronavirus disease-2019 (COVID-19), where inflammatory and immune-mediated mechanisms greatly contributed to disease progression [5]. Effective antiviral therapy may, therefore, require both control of viral replication and limitation of harmful host responses.

Direct-acting antivirals have transformed the outcome of several viral diseases by targeting essential viral proteins or enzymes [6]. However, their effectiveness may be limited by viral specificity, resistant variants, toxicity, and limited effects on host-mediated complications. These limitations strengthen investigation of complementary host-directed approaches that affect cellular processes involved in viral disease. Natural bioactive molecules with multi-target pharmacological properties are of particular interest, provided that their pharmacokinetic and delivery limitations can be addressed [7]. Thymoquinone (TQ; 2-isopropyl-5-methyl-1,4-benzoquinone), the principal bioactive constituent of *N. sativa* L. (black seed), is a potential candidate for such an approach. *N. sativa* has a long history of medicinal use, particularly for respiratory, inflammatory, and infectious conditions [8]. TQ is a small lipophilic quinone capable of interacting with intracellular signaling networks [9]. It modulates pathways involved in inflammation, oxidative stress, mitochondrial function, immune responses, and cell survival, properties that may be relevant to viral diseases involving both viral replication and host-mediated pathology [5].

Experimental studies have reported antiviral activity of TQ and *N. sativa*-derived preparations against different viruses, including hepatitis C virus (HCV), cytomegalovirus (CMV), and coronaviruses [5,10]. TQ also modulates host pathways implicated in viral immunopathology. It can suppress pro-inflammatory mediators, including tumor necrosis factor-alpha (TNF-α), interleukin-1 beta (IL-1β), and interleukin-6 (IL-6), and influence nuclear factor kappa-light-chain-enhancer of activated B cells (NF-κB), Signal Transducer and Activator of Transcription 3 (STAT3), and Interleukin-1 receptor-associated kinase 1 (IRAK1) signaling [11,12]. TQ also regulates redox homeostasis through the nuclear factor erythroid 2-related factor 2/Heme oxygenase-1 (Nrf2/HO-1) pathway and endogenous antioxidant defenses [13,14]. These virus-directed and host-modulating properties support further investigation of TQ in viral disease. However, its therapeutic development is restricted by poor aqueous solubility and low bioavailability [15,16]. These pharmaceutical limitations are discussed in detail in Section 3.

Nanotechnology provides a potential strategy to address these limitations. Liposomes, polymeric nanoparticles, solid lipid nanoparticles, nanostructured lipid carriers, nanoemulsions, and related systems can improve TQ solubility and enhance its pharmacokinetics [17]. More importantly for antiviral applications, nanocarriers may facilitate TQ delivery to the intracellular sites of viruses. However, improved systemic exposure or cellular uptake alone cannot establish antiviral superiority. Nano-TQ should ultimately demonstrate functionally relevant intracellular TQ exposure, and greater therapeutic benefit as compared to free TQ in viral infection models. The requirements for virus-informed intracellular delivery are discussed in Section 7.

In this review, the “dual antiviral concept” refers specifically to two complementary therapeutic dimensions: virus-directed activity that limits viral infection and replication, and host-protective activity that alleviates pathological host responses and virus-associated complications. Nanoformulation is considered a delivery strategy to augment these complementary actions. Accordingly, this review critically evaluates evidence for the virus-directed and host-protective effects of TQ, and examines the potential of nanotechnology to improve its therapeutic delivery. Particular attention is given to Nano-TQ platforms, functional intracellular delivery, virus-associated inflammatory and oxidative responses, associated complications, and translational development. We distinguish established evidence from emerging findings and propose nano-enabled mechanisms for translational objectives. Importantly, the therapeutic superiority of Nano-TQ over free TQ remains to be established experimentally.

## 2. Literature Search Strategy

A structured literature search was performed by using PubMed, Scopus, and Web of Science to identify relevant studies published from January 2000 to 2026. Only articles published in English were considered. The search included the terms “thymoquinone,” “*Nigella sativa*,” “thymoquinone nanoformulation,” “viral infection,” “antiviral activity,” “viral replication,” “intracellular drug delivery,” “nanocarrier trafficking,” “inflammation,” “oxidative stress,” “endothelial dysfunction,” “thromboinflammation,” “secondary infections,” and “host-directed therapy.” These terms were used alone or in relevant combinations. Reference lists of relevant articles were also examined to identify additional studies.

Studies were included if they provided experimental, clinical, pharmacological, computational, or mechanistic evidence relevant to TQ/*N. sativa* and viral infection, TQ nanoformulations, intracellular delivery, or host processes. Non-viral Nano-TQ studies were included when they provided useful information on formulation properties, pharmacokinetics, biodistribution, intracellular delivery, safety, or antimicrobial activity. Studies unrelated to the antiviral or Nano-TQ framework, or those providing insufficient relevant information, were excluded.

Evidence from purified TQ was distinguished from studies by using black seeds, oils, extracts, or combination preparations because these preparations cannot establish TQ-specific effects. Computational findings were considered mainly hypothesis-generating unless supported by experimental evidence. Greater emphasis was given to experimental and clinical studies stating virological, pharmacological, and disease-related outcomes. Because direct antiviral evidence for Nano-TQ remains limited, evidence from non-viral Nano-TQ reports was used mainly to evaluate its pharmaceutical and delivery potential, and not as proof of antiviral efficacy. This search was proposed to provide a structured and transparent basis for a narrative review and was not conducted as a formal systematic review or meta-analysis. Therefore, no formal risk-of-bias assessment or quantitative evidence synthesis was performed. During the preparation of this manuscript, the authors used ChatGPT (version GPT-4o) for the purposes of improving the spelling, grammar, and overall readability of the manuscript. The authors have reviewed and edited the output and take full responsibility for the content of this publication.

## 3. Physicochemical and Pharmacokinetic Barriers to Antiviral Delivery of TQ

TQ is a small, lipophilic monoterpene quinone derived predominantly from the volatile oil of *N. sativa*. Its substituted quinone ring, containing methyl and isopropyl groups, contributes to its lipophilicity, potentially facilitating interactions with lipid bilayers [18]. However, this hydrophobicity also results in poor aqueous solubility, limiting its reproducible formulation for systemic delivery.

Chemical stability represents an additional challenge. The redox-active quinone structure of TQ is sensitive to light, heat, and alkaline conditions, which may promote degradation during processing and storage [18]. Encapsulation within protective carriers may, therefore, improve aqueous dispersibility while reducing premature degradation. The pharmacokinetic behavior of free TQ further limits therapeutic exposure. Although its lipophilicity favors passive membrane diffusion, poor dissolution in the gastrointestinal environment can restrict oral absorption [19]. TQ also undergoes metabolic transformation and relatively rapid elimination. Reduction to thymohydroquinone and subsequent conjugation generate more hydrophilic metabolites that can be eliminated through biliary and renal routes [20]. Together, poor solubility, metabolism, and clearance can limit sustained exposure to free TQ. Biodistribution is also relevant to antiviral applications. Following systemic absorption, TQ primarily distributes to lipid-rich tissues such as the liver, kidneys, and lungs [15]. However, tissue exposure does not necessarily indicate effective delivery to virus-infected cells. The intracellular concentration and subcellular distribution of free TQ remain poorly defined. This is an important limitation because viral replication and several host responses involved in virus-associated pathology take place within specific intracellular environments.

Liposomes, lipid nanoparticles, polymeric nanoparticles, nanoemulsions, phospholipid-based systems, and related nanocarriers can improve TQ dispersibility and stability, resulting in superior pharmacokinetic and biodistribution profiles [17]. Pharmacokinetic studies with lipid-based TQ nanocarriers further indicate that nanoencapsulation can alter systemic exposure and tissue distribution as compared to free TQ [15]. For antiviral applications, however, improved bioavailability represents only one aspect of effective delivery. Nano-TQ systems would also need to provide sufficient TQ exposure inside infected cells and support appropriate intracellular release. Whether such formulations can achieve effective endosomal escape, intracellular retention, or preferential localization near viral replication compartments remains to be established experimentally. Thus, the major pharmaceutical barriers of TQ, such as poor aqueous solubility, chemical instability, metabolism, limited systemic exposure, and poorly defined intracellular distribution, provide key considerations for Nano-TQ design. Nanoengineering may, therefore, extend beyond improving bioavailability toward obtaining relevant intracellular delivery at the virus–host interface (Figure 1).

## 4. Nano-TQ as an Emerging Antiviral Platform: Bridging Antiviral Activity and Intracellular Delivery

The reported antiviral activity of TQ provides a basis for exploring its development as an antiviral agent. One of the clearest examples of direct TQ-specific activity comes from Chikungunya virus (CHIKV). TQ was shown to bind a conserved hydrophobic pocket of the viral capsid protein involved in capsid assembly and budding. In cell-based experiments, TQ inhibited CHIKV replication with an EC_50_ of 4.478 μM and reduced viral RNA levels [21]. These outcomes support a potential virus-directed mechanism in CHIKV, although its consequence for other alphaviruses remains to be established. Evidence from other viral systems is supportive but less direct. In murine CMV infection, *N. sativa* oil reduced viral burden in organs (liver and spleen), and was associated with enhanced antiviral immune responses [22]. Experimental and computational studies in Severe Acute Respiratory Syndrome Corona Virus-2 (SARS-CoV-2) models have suggested that TQ may interfere with processes associated with viral entry [23,24], while computational analyses have proposed interactions with SARS-CoV-2-related targets [25,26]. These findings broaden the possible antiviral relevance of TQ and *N. sativa* but require cautious interpretation. The strength of this evidence depends on the intervention and study design. Studies using purified TQ provide the most direct evidence of TQ-specific activity, whereas findings from *N. sativa* seeds, oils, extracts, or combination preparations cannot be attributed exclusively to TQ. Similarly, computational studies can identify plausible molecular interactions but remain primarily hypothesis-generating until experimentally validated. Further studies using purified TQ, relevant infection models, and quantitative virological endpoints are therefore needed to establish virus-specific antiviral effects.

Limited clinical observations with *N. sativa*-based interventions have also been reported, although their methodological limitations impede conclusions regarding TQ-specific antiviral ability. In patients with HCV, *N. sativa*-based interventions were associated with a decline in viral load and improvements in selected liver-related parameters [27,28]. However, these studies used whole *N. sativa* preparations, either alone or in combination with other natural products, making it difficult to attribute the noted effects specifically to TQ. Evidence in HIV is considerably weaker and is limited to individual case reports depicting reduced viral load and improved CD4^+^ T-cell responses following *N. sativa*-based interventions [29,30]. The absence of randomization, sufficient sample size, and standardized TQ exposure substantially limits causal understanding of these observations. These findings should, therefore, be considered preliminary and hypothesis-generating rather than clinical evidence of antiviral efficacy of TQ. Controlled clinical studies by using standardized and chemically characterized TQ formulations are required to establish its clinical antiviral potential.

Nanoformulations add a potentially important but less established dimension. TQ nanocarriers have shown improvements in formulation stability, solubility, pharmacokinetics, and therapeutic performance in several experimental settings, but direct evidence that Nano-TQ provides greater antiviral efficacy than free TQ remains limited. Improved pharmaceutical performance should therefore not be considered evidence of antiviral superiority. Direct comparative studies should evaluate free TQ and Nano-TQ by using virological and pharmacological endpoints, including viral RNA and protein expression, infectious viral output, therapeutic selectivity, and intracellular TQ exposure, to determine whether nanoencapsulation provides a reproducible antiviral advantage.

Accordingly, Nano-TQ should presently be regarded as an emerging antiviral delivery strategy rather than an established antiviral nanomedicine. Its development is supported by the pharmacological properties of TQ and the potential pharmaceutical advantages of nanoformulation, but its added therapeutic value requires experimental demonstration. A key benchmark should be greater antiviral efficacy than free TQ at safe and clinically relevant exposures. The available evidence therefore ranges from direct studies with purified TQ to supportive *N. sativa*-based findings and proposed Nano-TQ applications, which represent different levels of experimental and translational certainty (Table 1).

## 5. TQ at the Virus–Host Interface: Antiviral and Host-Directed Mechanisms

Mechanistic evidence can be considered at three levels: effects demonstrated during viral infection, TQ pharmacological effects from other inflammatory or oxidative models that are relevant to viral pathology, and proposed Nano-TQ-mediated therapeutic effects that require experimental validation. Beyond potential effects on viral replication, TQ may alter host pathways implicated in disease progression. Viral infections can lead to inflammation and immunopathology that can contribute to tissue injury and complications [31]. Because TQ can influence multiple signaling and cytoprotective pathways, it may provide complementary virus-directed and host-protective actions. These effects should remain distinct: virus-directed activity may contribute to viral control, whereas host modulation may primarily reduce infection-associated complications.

### 5.1. Virus-Directed Activity and Potential Interference with Viral Processes

As discussed in Section 4, experimental evidence indicates that TQ can decrease viral replication, although the strength and specificity of this evidence vary among viruses. TQ may intervene directly with selected viral processes or modify host–cell conditions essential for viral propagation. Computational studies have proposed additional interactions with viral molecules involved in viral entry and replication, but these remain hypothesis-generating until experimentally substantiated. Because viruses diverge in their entry pathways, replication strategies, and host-factor requirements, a uniform antiviral mechanism should not be anticipated. Future studies should identify the stages of viral life-cycle impacted by TQ and distinguish direct interactions with viral components from effects on host physiological processes.

### 5.2. Regulation of Virus-Induced Inflammatory Signaling

Inflammatory signaling plays an essential role in antiviral defense, but excessive or persistent activation can contribute to virus-associated tissue injuries [3,32]. Viral recognition promotes cytokine and chemokine production that is needed for immune-cell recruitment and viral control. However, unresolved inflammatory responses may contribute to endothelial injury, vascular dysfunction, and systemic complications. TQ can modulate several inflammatory pathways, including NF-κB, and reduce inflammatory mediators such as TNF-α, IL-1β, and IL-6. The effects of TQ on IRAK1, STAT3, nitric oxide, and prostaglandin production have also been reported [11,12]. These actions suggest that TQ may alter inflammatory amplification at multiple levels. In viral disease, this could potentially help in limiting excessive inflammation. However, because innate inflammatory pathways also help in viral clearance, the therapeutic objective should be the modulation of dysregulated inflammation, while preserving protective antiviral immunity.

### 5.3. Redox and Mitochondrial Control at the Virus–Host Interface

Oxidative stress and mitochondrial dysfunction offer another link between viral infection and host injury [33]. Viral infection can increase reactive oxygen species (ROS) and disturb mitochondrial function, while oxidative stress and inflammation may boost each other and contribute to tissue damage. TQ may influence this process via its antioxidant and cytoprotective actions. Experimental studies indicate that TQ can reduce oxidative stress and support endogenous defenses, including superoxide dismutase (SOD), catalase, glutathione peroxidase (GPx), and reduced glutathione (GSH) [13,14]. TQ has also been associated with Nrf2/HO-1 signaling and preservation of mitochondrial integrity and redox homeostasis [34]. These effects may help in modulating excessive ROS accumulation and redox-sensitive inflammatory signaling. However, the specific contribution of Nrf2/HO-1 activation and mitochondrial protection to antiviral effects remains insufficiently defined and requires virus-specific validation.

### 5.4. Convergence of Virus-Directed and Host-Protective Actions: Rationale for Nano-TQ

The therapeutic potential of TQ may result from complementary actions at the virus–host interface. TQ may interfere with selected viral or virus-dependent processes while modulation of inflammatory, oxidative, mitochondrial, and immune responses may help limit host-mediated pathology. Viral control and host protection are related but distinct outcomes, and their relative importance is likely to depend on the virus, infected tissue, disease severity, and stage of infection. This dual-action profile provides a rationale for investigating Nano-TQ as a delivery strategy that may improve the therapeutic expression of TQ pharmacology [10,35]. However, improved formulation performance should not be considered evidence of enhanced antiviral efficacy. Any advantage should be demonstrated by comparing Nano-TQ with free TQ by using infectious viral output and relevant measures of host pathology. Thus, Nano-TQ may potentially enhance complementary virus-directed and host-protective effects, but this remains to be established experimentally. The formulation and intracellular-delivery requirements are discussed in subsequent sections, while Figure 2 summarizes the proposed dual-action framework and its potential integration with established antiviral therapy.

## 6. Engineering TQ Nanoformulations for Antiviral Delivery

Nanoformulation offers a practical strategy to address the pharmaceutical limitations of TQ described in Section 3 by improving its stability, pharmacokinetics and tissue distribution. For antiviral applications, however, these improvements are meaningful only if they translate into greater exposure at disease-relevant sites and improved therapeutic outcomes. Several TQ delivery platforms, including liposomes, solid lipid nanoparticles (SLNs), nanostructured lipid carriers (NLCs), polymeric nanoparticles, nanoemulsions, and other phospholipid- or lipid-based systems, have been investigated [17]. Nanoencapsulation has improved several pharmaceutical properties of TQ, while TQ-loaded NLCs have demonstrated altered pharmacokinetics and biodistribution following oral and intravenous administration [15]. However, direct evidence linking these improvements to superior antiviral efficacy remains limited and should be considered when evaluating individual Nano-TQ platforms.

### 6.1. Liposomal TQ: A Versatile Platform for Antiviral Delivery

Liposomes are well suited to TQ because their phospholipid bilayers accommodate lipophilic compounds while permitting dispersion in aqueous biological environments [36]. Encapsulation can protect TQ from degradation and improve its solubility and biodistribution [37]. Particle size, lipid composition, surface charge, membrane properties, and surface functionalization can also be adjusted according to the intended route and tissue target [38]. For antiviral applications, liposomal TQ could potentially facilitate local delivery to affected tissues. Pulmonary delivery may be relevant to respiratory infections, while liposomes also offer opportunities for combination therapy. Co-administration with an established antiviral represents a relatively practical strategy, whereas co-encapsulation could be considered when synchronized delivery provides a demonstrable therapeutic advantage. These approaches require comparison with free TQ and standard antiviral therapy.

### 6.2. SLNs and NLCs: Exploiting the Lipophilic Nature of TQ

SLNs and NLCs are well suited to lipophilic TQ [39]. Their lipid matrices can improve aqueous dispersibility, protect against degradation, and provide sustained release. NLCs may additionally improve drug loading and reduce drug expulsion during storage. Pharmacokinetic studies show that TQ-loaded NLCs can improve systemic exposure and tissue distribution following oral and intravenous administration [15,40]. These findings demonstrate that TQ disposition may alter, but do not establish preferential delivery to infected tissues or superior antiviral activity. Future studies should therefore relate these pharmacokinetic advantages to disease-relevant tissue exposure and measurable virological outcomes.

### 6.3. Polymeric Nanoparticles: Controlled and Sustained TQ Delivery

Biodegradable polymeric nanoparticles provide a versatile approach for controlled TQ delivery. Poly(lactic-co-glycolic) acid (PLGA), Polyethylene Glycol (PEG)-containing polymers, PEG-PLA, and chitosan-based systems can improve TQ stability and regulate release kinetics of the drug [41]. Their surfaces can also be modified to influence circulation, tissue distribution, and interactions with certain cell populations. Surface ligands may potentially support cell-directed delivery, although selective targeting of virus-infected cells remains unproven for Nano-TQ. Chitosan-based systems may be particularly beneficial for mucosal administration because of their mucoadhesive characteristics [42]. Thus, polymeric systems offer tunable release and surface characteristics, but their functional antiviral value demands direct experimental validation.

### 6.4. Nanoemulsions and Oral Nano-TQ: Improving Systemic Exposure

Nanoemulsions and related lipid-based systems can improve the solubilization and gastrointestinal absorption of hydrophobic compounds such as TQ [43]. Preserving TQ in a dispersed lipid environment may enhance oral exposure and reduce variability associated with conventional formulations. Oral Nano-TQ may therefore be suitable for repeated administration, chronic infections, or adjunctive therapy. Its major practical advantages are non-invasive administration and probable suitability for extended treatment. However, enhanced oral exposure should finally be linked to therapeutically relevant tissue concentrations and measurable disease-related outcomes.

### 6.5. Inhalable Nano-TQ: A Potential Future Strategy for Respiratory Viral Infections

Pulmonary delivery represents a potential future approach for respiratory viral infections. The majority of respiratory viruses multiply within airway or alveolar epithelial cells, while severe disease may involve macrophage activation, oxidative stress, inflammation, and alveolar–vascular injury [44]. Local administration could potentially increase TQ exposure at pulmonary locations while limiting systemic distribution. However, direct in vivo evidence for antiviral efficacy of inhaled Nano-TQ is presently lacking, and this approach should therefore be considered prospective.

Liposomes and selected polymeric carriers may be adaptable to aerosol delivery, consistent with broader nanoparticle-based approaches being investigated for respiratory viral infections [45]. Development should consider aerosol stability, lung deposition, pulmonary retention, cellular uptake, and TQ stability during aerosolization or nebulization. Future studies should link these pharmaceutical parameters with pulmonary TQ exposure, quantitative antiviral endpoints, inflammatory responses, lung pathology, and repeated-dose safety. Such evidence is required before inhalable Nano-TQ can progress toward translational development.

### 6.6. From Pharmaceutical Optimization Toward Functional Antiviral Delivery

Beyond conventional formulation characterization, Nano-TQ should be evaluated according to whether improved pharmaceutical performance produces greater antiviral or host-protective effects than free TQ at comparable or safe doses. Particle characteristics, drug loading, release, pharmacokinetics, and biodistribution remain important, but none alone demonstrates antiviral target engagement or therapeutic superiority. Evaluation should therefore progress from formulation performance → relevant tissue exposure → cellular TQ availability → antiviral and host-protective effects. Detailed requirements for intracellular trafficking, subcellular localization, and functional antiviral delivery are discussed in Section 7.

### 6.7. Next-Generation Nano-TQ: Toward Virus-Informed Nanocarrier Design

Advances in antiviral nanomedicine provide design principles for improving targeted delivery, intracellular release, and therapeutic performance. Future Nano-TQ systems could incorporate advanced features when conventional carriers cannot provide sufficient exposure [46]. Surface functionalization may enhance delivery to disease-relevant cells, while pH-, membrane-, or stimuli-responsive systems may provide greater control over TQ release [47,48,49]. ROS-responsive systems are also of interest because increased oxidative stress in infected or inflamed tissues could potentially provide a trigger for localized release [50].

These approaches remain largely prospective for Nano-TQ and should be introduced only when supported by a clear biological need. Carrier selection, surface modification, release mechanism, and administration route should be guided by the targeted virus, affected tissue, and disease stage. Detailed intracellular and subcellular targeting strategies are discussed in Section 7. Overall, existing evidence shows that lipid-, polymer-, and emulsion-based systems can improve the pharmaceutical properties of TQ, whereas their antiviral advantage remains insufficiently established. The evidence can therefore be considered at three levels: experimentally established effects of free TQ, pharmaceutical advantages of Nano-TQ demonstrated predominantly in non-viral models, and proposed antiviral advantages requiring direct validation (Table 2).

## 7. Potential Intracellular Delivery of Nano-TQ: From Cellular Uptake to Functional Antiviral Delivery

Nanoformulation can improve the physicochemical and pharmacokinetic properties of TQ, including its stability, systemic exposure, and tissue distribution. However, these established advantages should be distinguished from proposed intracellular targeting capabilities. Direct evidence that Nano-TQ selectively reaches endosomes, the cytosol, ER-associated viral replication sites, the nucleus, or mitochondria during viral infection is currently lacking. Most compartment-specific strategies discussed here are therefore based on broader principles of intracellular nanomedicine and represent future engineering opportunities rather than demonstrated properties of Nano-TQ (Table 3).

Intracellular delivery, nevertheless, remains important because viruses exploit distinct cellular compartments for entry, replication, assembly, and release, while infection can unsettle inflammatory and mitochondrial pathways [50]. Thus, nanoparticle uptake alone does not demonstrate effective antiviral delivery. A more meaningful benchmark is an effective intracellular delivery, in which TQ is released in an active form, reaches a relevant virus–host site, and produces a measurable antiviral or host-protective response. The following discussion therefore provides an evidence-informed framework for virus-specific Nano-TQ engineering.

### 7.1. Virus-Informed Intracellular Delivery: A Future Engineering Framework

Viruses differ substantially in their intracellular life cycles, with relevant processes occurring within endosomes, the cytosol, membrane-associated replication structures, or the nucleus [51]. Nano-TQ design should therefore align nanocarrier properties and intracellular release while also considering the biology of the targeted virus. Broader nanomedicine studies suggest that pH-responsive lipids, polymers, and peptides can facilitate endosomal release or cytosolic delivery, while fusogenic systems can promote membrane interaction and endosomal escape. Cell-penetrating peptides may increase cellular uptake, although endosomal retention can remain limiting. Specialized approaches have also been investigated for nuclear and mitochondrial delivery [52,53].

Accordingly, controlled endosomal release could be explored for entry or uncoating of viruses within endocytic compartments, whereas cytosolic delivery may be more relevant to cytoplasmic or membrane-associated replication. Organelle-directed approaches could also be considered where specific intracellular host pathways contribute to viral pathogenesis. These potential relationships are illustrated in Figure 3 and summarized in Table 3. Importantly, these strategies remain largely conceptual for antiviral Nano-TQ. Preferential localization at viral replication sites using pH-sensitive, fusogenic, cell-penetrating peptide-functionalized, or organelle-directed systems has not been demonstrated in virus-specific models. Future studies should determine whether such modifications alter intracellular TQ distribution and improve target engagement, infectious viral output, or host protection as compared to free TQ and conventional Nano-TQ.

**Table 3:** Virus-informed mapping of intracellular viral processes to potential Nano-TQ delivery strategies.

Intracellular Viral Process or Therapeutic Requirement	Potential Future Nanocarrier Strategy	Intended Delivery Rationale	Current Evidence for Nano-TQ
Endosomal viral entry or uncoating [47,48]	pH-responsive release or controlled endosomal retention	Promote TQ release within acidic endosomal compartments where relevant viral processes occur	Hypothetical
Cytosolic viral replication [47,52]	pH-sensitive, endosomolytic, or fusogenic carriers	Promote endosomal escape and increase cytosolic TQ availability	Hypothetical
Membrane-associated viral replication complexes [47,52]	Controlled cytosolic release or membrane-interacting lipid systems	Improve TQ availability near cytoplasmic membrane-associated replication sites	Hypothetical
Potential nuclear-directed ligands [52,53]	Potential nuclear-directed ligands	Facilitate intracellular trafficking toward the nucleus where relevant viral processes occur	Hypothetical
Virus-associated mitochondrial dysfunction [47,52]	Potential mitochondria-directed carrier design	Increase TQ exposure near mitochondrial pathways involved in oxidative stress and cellular injury	Hypothetical

This mapping should therefore be viewed as a roadmap for future Nano-TQ engineering rather than evidence of established intracellular targeting. Experimental studies should determine whether these carrier modifications increase TQ exposure within the intended intracellular compartment and whether this translates into target engagement and reduced infectious viral output.

### 7.2. From Nanoparticle Internalization to Functional Intracellular Delivery

Nanoparticle uptake should be distinguished from functional drug delivery. Many nanocarriers enter cells through endosomal pathways and may subsequently undergo lysosomal processing [54]. High nanoparticle uptake therefore does not ensure that pharmacologically active TQ reaches the intended viral or host target.

For Nano-TQ, a useful sequence is: cellular uptake → intracellular TQ release → appropriate subcellular localization → target engagement → biological response. The required trafficking behavior depends on the intended target. Endosomal escape may be important for cytosolic targets, whereas endosomal retention could be useful when relevant virus–host processes occur within these compartments. Future studies should therefore move beyond fluorescence-based nanoparticle uptake and quantify intracellular TQ itself. Where feasible, TQ exposure and subcellular localization should be related to viral RNA or DNA, viral protein expression, and particularly infectious viral output. Such exposure–response relationships would provide stronger evidence of functional intracellular delivery.

### 7.3. Targeting Infected and Inflammatory Cells

Intracellular Nano-TQ delivery may also be relevant to pathological host responses. Infected epithelial cells and activated immune cells can promote inflammation and tissue injury through NF-κB, IRAK1, STAT3, inflammasome signaling, and oxidative stress pathways [55,56]. As discussed in Section 5, TQ can modulate several of these pathways, suggesting that increased exposure within disease-relevant cells could potentially improve host-protective effects. General nanocarrier biology suggests that macrophages, monocytes, and epithelial cells may represent potential cellular targets. However, uptake by these cells should not be interpreted as selective targeting of infected cells. Such selectivity would require direct comparison of Nano-TQ distribution between infected and uninfected populations. Furthermore, the modulation of inflammatory cells should curtail pathological immune activation without compromising antiviral defense; inflammatory outcomes should therefore be evaluated alongside viral clearance.

### 7.4. Linking Functional Intracellular Delivery to Host Protection

The relevance of intracellular Nano-TQ delivery ultimately depends on whether cellular effects translate into protection from virus-associated pathology. Viral infection can promote oxidative imbalance, mitochondrial dysfunction, inflammatory amplification, and tissue injury. Delivery of TQ to relevant cells could potentially influence these processes through the antioxidant, mitochondrial, and inflammation-regulating activities described in Section 5. However, an improved intracellular exposure alone does not establish protection against acute lung injury, endothelial dysfunction, cardiovascular complications, or secondary infections. Such benefits require direct evidence in appropriate infection models. Evidence concerning specific virus-associated complications is therefore considered separately in Section 8.

### 7.5. Establishing Proof of Intracellular Antiviral Targeting

A major challenge is to establish whether Nano-TQ achieves greater antiviral efficacy specifically through improved cellular and subcellular TQ exposure. Current evidence supports several pharmacological activities of TQ and demonstrates that nanoformulation can improve its pharmaceutical and pharmacokinetic properties [57]. A stronger demonstration of functional targeting would establish the sequence: tissue delivery → disease-relevant cell uptake → intracellular TQ release → subcellular localization → target engagement → reduced infectious viral output → reduced tissue injury → improved disease outcome. Nano-TQ should therefore be compared directly with free TQ at appropriately matched exposures and, where relevant, with standard antiviral therapy. Fluorescent or isotope-based carrier tracking, and quantitative intracellular TQ measurement could define spatial distribution, but these findings should ultimately be supported by improved disease outcomes. The central translational question is whether nanoformulation can transform TQ pharmacology into reproducible therapeutic advantage. Demonstrating this relationship would provide stronger evidence for advancing Nano-TQ toward functionally targeted antiviral therapy.

## 8. Nano-TQ Against Virus-Associated Complications: From Host-Response Modulation to Tissue Protection

The severity of viral infections is determined not only by viral replication but also by pathological host responses [58]. Persistent inflammation, oxidative and mitochondrial stress, endothelial activation, and coagulation disturbances can contribute to pulmonary, cardiovascular, neurological, and systemic complications [59]. Because TQ modulates several of these processes, Nano-TQ may have potential as a host-protective adjunct by improving TQ exposure within infected and inflamed tissues. However, these potential benefits remain to be demonstrated in virus-specific models.

### 8.1. Cytokine Storm and Dysregulated Host Immunity

Effective antiviral immunity requires coordinated cytokine and chemokine responses, but dysregulation of these processes has been associated with severe SARS, MERS, COVID-19, influenza, adenovirus, and respiratory syncytial virus (RSV) infections [60,61]. The magnitude and timing of these responses vary according to the virus and host factors, including age, comorbidities, coagulopathy, and secondary infections. COVID-19 provides a well-characterized example. Longitudinal studies have identified persistent shifts in IL-1β, IL-1Ra, TNF-α, IL-6, IL-2, IL-8, and IL-18, together with disturbances in antiviral interferon responses [3,62]. IL-6, IL-2, IL-1Ra, and IL-18 have shown their role in disease severity, while sustained IL-2, IL-8, and IL-1Ra were associated with progression toward critical illness [63]. Such imbalance may contribute to acute respiratory distress syndrome (ARDS), vascular and coagulation abnormalities, and multiorgan injury [63]. These findings support the restoration of the balance between protective antiviral immunity and pathological inflammation rather than generalized immunosuppression. TQ can modulate inflammatory signaling and reduce several pro-inflammatory mediators [64]. Nanoformulation could potentially improve its exposure within inflamed tissues and thereby support modulation of excessive cytokine responses. Future studies should simultaneously assess cytokine profiles, viral clearance, protective immune functions, and tissue injury to determine whether Nano-TQ can limit pathological inflammation.

### 8.2. Oxidative Stress and Acute Tissue Injury

Oxidative stress can amplify inflammation-associated tissue injury [65]. Viral infection and activated immune cells may increase ROS, impair mitochondrial function, and weaken antioxidant defenses, creating an interconnected inflammation–oxidative stress–tissue injury axis [31]. TQ may potentially influence this process through its antioxidant and cytoprotective activities, while Nano-TQ could improve exposure within affected tissues. This concept may be particularly relevant to viral acute lung injury (ALI) and ARDS, where epithelial injury, oxidative stress, inflammatory-cell infiltration, and disruption of the alveolar–vascular barrier impair pulmonary function [66]. Nano-TQ could potentially combine virus-directed and tissue-protective effects within the infected lung, particularly through local pulmonary delivery. However, protection against viral ALI/ARDS remains unproven and should be evaluated using integrated virological, inflammatory, histopathological, and respiratory endpoints.

### 8.3. Virus-Induced Endothelial Dysfunction, Thromboinflammation, and Multiorgan Injury

Severe viral infections can disrupt vascular homeostasis through sustained inflammation, endothelial activation, and altered interactions among monocytes, platelets, and coagulation pathways [55,56]. In severe dengue, infected monocytes can release viral protein-containing microparticles that promote a proinflammatory and procoagulant endothelial phenotype, and contribute to vasculopathy [67]. Studies indicated that viral infections, including influenza, SARS-CoV-2, HIV, HCV, and herpes zoster, are associated with increased cardiovascular risk [68]. In severe systemic and respiratory viral disease, inflammatory signals may promote endothelial dysfunction, tissue factor expression, platelet activation, and impaired anticoagulant regulation [69]. The resulting thromboinflammatory and microvascular insults can contribute to pulmonary, cardiovascular, renal, and multiorgan complications. In this setting, the potential relevance of TQ lies primarily in modulating inflammatory and oxidative signals that promote vascular dysfunction rather than acting as a direct anticoagulant or fibrinolytic agent. Nano-TQ could potentially improve TQ distribution within inflamed tissues and thereby limit progression along the virus–inflammation–endothelial dysfunction–thromboinflammation axis. This integrated hypothesis remains to be tested. Future studies should determine whether changes in viral burden are accompanied by reduced inflammation, endothelial activation, coagulation abnormalities, and organ pathology. This proposed progression is summarized in Figure 4.

### 8.4. Secondary Microbial Infections

Secondary bacterial and fungal infections are important complications of severe viral disease, particularly in the presence of epithelial injury, immune dysregulation, prolonged hospitalization, or other predisposing factors [70]. Among 253 critically ill COVID-19 patients, 83 (32.8%) developed fungal coinfections, including candidiasis (24.1%), aspergillosis (4.3%), and mucormycosis (1.97%); poor diabetes control, corticosteroid exposure, and multiple comorbidities were important risk factors [71]. In another study of 122 COVID-19 patients with secondary infections, *Klebsiella pneumoniae* and *Acinetobacter baumannii* were predominant isolates, with increased carbapenem resistance also reported [72]. These observations emphasize the importance of microbiological surveillance, infection control, and antimicrobial stewardship.

The antibacterial and antifungal activities of TQ and its nanoformulations provide a rationale for investigating their potential in this setting. TQ-loaded liposomes have demonstrated therapeutic activity against drug-resistant *A. baumannii* and virulence-associated processes of *C. albicans* [39,73,74]. However, these findings were not obtained in viral coinfection models and therefore do not establish efficacy against secondary infections during viral disease. Sequential viral–bacterial and viral–fungal models could determine whether Nano-TQ reduces secondary microbial burden while preserving antiviral immunity and controlling viral infection. Combination with standard antibacterial or antifungal agents also warrants investigation. More broadly, Nano-TQ could potentially influence interconnected stages of viral disease involving viral infection, dysregulated host responses, tissue and vascular injury, and secondary microbial complications. Establishing such multifunctional benefit will require integrated models assessing viral and microbial burdens, host immunity, tissue pathology, and organ function.

## 9. Translating Nano-TQ into Antiviral Therapy: From Experimental Promise to Clinical Development

Despite its pharmacological rationale, Nano-TQ remains at an early stage of antiviral translation. Current evidence indicates that TQ has biological activities relevant to viral disease and that nanoformulation can improve its pharmacokinetic properties. The critical next step is to determine whether these advantages produce superior antiviral efficacy and clinically relevant protection at safe and achievable exposures. Development should therefore focus on relationships among Nano-TQ exposure, antiviral activity, disease outcomes, and safety.

### 9.1. Nano-TQ in the Context of Conventional Antiviral Therapy

Approved antiviral drugs remain the clinical benchmark for evaluating Nano-TQ. Direct-acting antivirals can provide potent, virus-specific suppression, and clinical efficacy is established through preclinical and clinical studies. In contrast, Nano-TQ remains experimental, and direct comparisons with approved antivirals are insufficient to establish comparable potency. Nano-TQ should therefore not presently be considered an alternative to clinically validated antiviral therapy. Key differences in potency, therapeutic index, mechanism, clinical evidence, accessibility, and cost are summarized in Table 4. The potential value of Nano-TQ may instead lie in its complementary therapeutic profile. Whereas direct-acting antivirals primarily suppress defined stages of the viral life cycle, Nano-TQ could potentially combine TQ delivery with modulation of pathological host responses involving inflammation, oxidative stress, and tissue injury. This provides a rationale for investigating Nano-TQ as an adjunct to established antivirals, provided that additional benefit can be demonstrated without compromising antiviral efficacy. Accessibility and cost also require careful evaluation. Although TQ is derived from a widely available natural source, nanocarrier production, characterization, and quality control may increase costs. Because validated manufacturing and health-economic data for antiviral Nano-TQ are unavailable, claims of greater affordability or accessibility remain premature. Future comparisons should therefore consider antiviral potency, therapeutic index, safety, dosing, accessibility, and cost-effectiveness.

### 9.2. Bridging the Preclinical-to-Clinical Evidence Gap

The therapeutic role of Nano-TQ is likely to differ with the targeted virus and disease stage. Antiviral activity may be more relevant during replication-dominant phases, whereas host-protective effects may become increasingly important during persistent inflammation, endothelial dysfunction, or thromboinflammation. Development should therefore define the intended viral infection, disease stage, patient population, administration route, and therapeutic role rather than assume a uniform application. A major preclinical priority is to establish whether Nano-TQ provides a meaningful advantage over free TQ in viral models [57]. Studies should progress from infected-cell and advanced tissue models to appropriate animal models and directly compare free TQ, Nano-TQ, blank nanocarrier, and standard antiviral therapy. Virological endpoints, particularly infectious viral output, should be integrated with tissue pathology and clinically relevant measures of disease severity. For adjunctive use, combination partners should be selected mechanistically, and studies should evaluate pharmacodynamic interactions, toxicity, and whether co-administration provides greater benefit than either intervention alone.

Pharmacokinetic–pharmacodynamic (PK–PD) studies are essential to define exposure–response relationships and guide dose selection [75]. TQ exposure in infected tissues and, where feasible, disease-relevant cells should be related to antiviral and pathological outcomes rather than relying solely on plasma concentrations. Administration route and treatment schedule should also reflect the clinical application: pulmonary delivery may suit respiratory infections, oral administration may support outpatient or prolonged treatment, and parenteral administration may provide rapid systemic exposure. Treatment timing should similarly reflect whether viral control or modulation of pathological host responses is the dominant objective.

### 9.3. Safety, Pharmaceutical Development, and Clinical Readiness

Clinical translation requires Nano-TQ to be a well-defined, reproducible, and acceptable pharmaceutical product. Safety studies should distinguish TQ-related toxicity from effects of the nanocarrier and interactions with antiviral drugs. Inhalable formulations will additionally require evaluation of pulmonary tolerability, particle clearance, and repeated aerosol exposure. Nanoencapsulation should ultimately maintain an acceptable therapeutic window relative to free TQ.

Manufacturing feasibility should also be addressed early. Critical quality attributes include particle size and distribution, TQ loading, release behavior, and batch-to-batch reproducibility. The preferred formulation should be the simplest scalable platform capable of achieving the required exposure and efficacy. Early clinical studies should first establish safety and human pharmacokinetics before evaluating appropriate clinical outcomes. The key translational benchmark is whether Nano-TQ provides a reproducible therapeutic advantage over free TQ at safe and clinically achievable doses. The overall pathway, from antiviral pharmacology to PK–PD optimization, safety, scalable manufacturing, and clinical evaluation, is summarized in Figure 5.

## 10. Future Directions: From Nanoformulation to Precision Antiviral Nanomedicine

Future Nano-TQ research should focus on whether nanoengineering provides a meaningful antiviral advantage rather than further demonstrating pharmaceutical improvement alone. Physiologically relevant systems, including advanced cell models, air–liquid interface respiratory models, and appropriate animal infection models, should directly compare free TQ, Nano-TQ, standard antivirals, and relevant combinations by using disease-related endpoints. Spatial pharmacology will be particularly important for determining whether TQ reaches infected cells and relevant intracellular sites rather than simply accumulating within an affected organ. Fluorescent or isotope-based tracking, quantitative intracellular TQ measurements, subcellular fractionation, and imaging could link TQ localization with antiviral activity. Where justified by such evidence, ligand-targeted, endosomal escape-promoting, or organelle-directed systems could then be investigated. Claims of infected-cell or subcellular targeting should remain prospective until directly demonstrated. Nano-TQ development should also become virus-informed rather than formulation-driven. Differences in viral tropism, intracellular replication, disease kinetics, and immunopathology are likely to require different delivery strategies. Respiratory infections may benefit from local pulmonary delivery, whereas systemic or persistent infections may require sustained systemic exposure. Treatment timing should likewise reflect whether viral replication or host-mediated inflammatory and thromboinflammatory injury is the dominant therapeutic target.

Clinical translation will require rigorous pharmacokinetic, safety, manufacturing, and regulatory evaluation. Candidate formulations should demonstrate reproducible particle characteristics, TQ release, stability, batch consistency, and scalable production. PK–PD studies should define exposure–response relationships and therapeutic windows, while safety assessments should consider TQ, the nanocarrier, administration route, repeated exposure, tissue accumulation, immunological compatibility, and interactions with concomitant antiviral drugs.

Accordingly, the major priorities are to establish whether Nano-TQ outperforms free TQ in virus-specific models, determine whether any advantage is linked to intracellular TQ exposure and target engagement. Progress toward clinical evaluation should depend on therapeutic benefit at safe doses. The broader translational pathway is summarized in Figure 5. This review has several limitations. As a narrative and concept-driven review rather than a systematic review or meta-analysis, its literature coverage may not be exhaustive, and formal assessment of study quality or risk of bias was not performed. Direct antiviral evidence for purified TQ is heterogeneous, while studies specifically evaluating Nano-TQ in viral infection models remain limited. Consequently, some proposed Nano-TQ applications are informed by *N. sativa*-based interventions, non-viral Nano-TQ studies, or mechanistic evidence from related disease models. These indirect findings should not be interpreted as established antiviral efficacy. Further virus-specific studies directly comparing Nano-TQ with free TQ and standard antiviral therapy are required to validate the proposed concepts.

## 11. Conclusions

TQ has potential virus-directed and host-protective activities, while nanoformulation may improve its pharmaceutical properties and delivery to disease-relevant tissues and cells. However, the antiviral value of Nano-TQ cannot be established from improved bioavailability or cellular uptake alone. Direct evidence demonstrating functionally relevant intracellular TQ exposure, target engagement, and greater therapeutic benefit than free TQ remains limited. Future studies should therefore prioritize virus-specific comparative models, quantitative PK–PD and spatial pharmacology, safety, and scalable formulation development. Nano-TQ should presently be regarded as an emerging antiviral delivery strategy rather than an established antiviral therapy. Demonstrating a reproducible therapeutic advantage at safe and clinically achievable exposures will be essential for its progression toward clinical application.

## Figures and Tables

**Figure 1:**
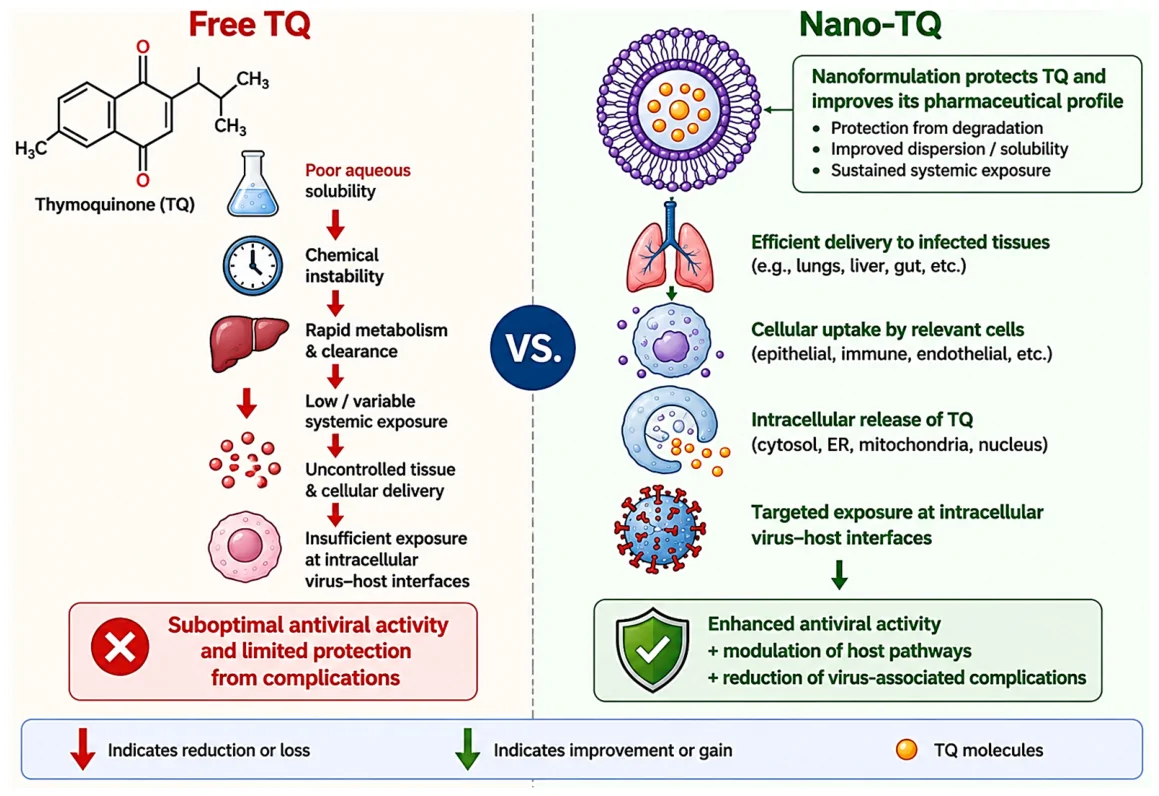
Why Nano-TQ? From the delivery barrier to the intracellular antiviral opportunity. Free TQ is limited by poor aqueous solubility, rapid metabolism and cellular exposure, which may restrict its availability at intracellular virus–host interfaces. Nanoformulation may help TQ to overcome these limitations by improving stability, sustaining exposure, and facilitating delivery to infected tissues. These properties could potentially enhance antiviral activity while also supporting modulation of host-mediated injury. The proposed intracellular antiviral advantages of Nano-TQ require further experimental validation.

**Figure 2:**
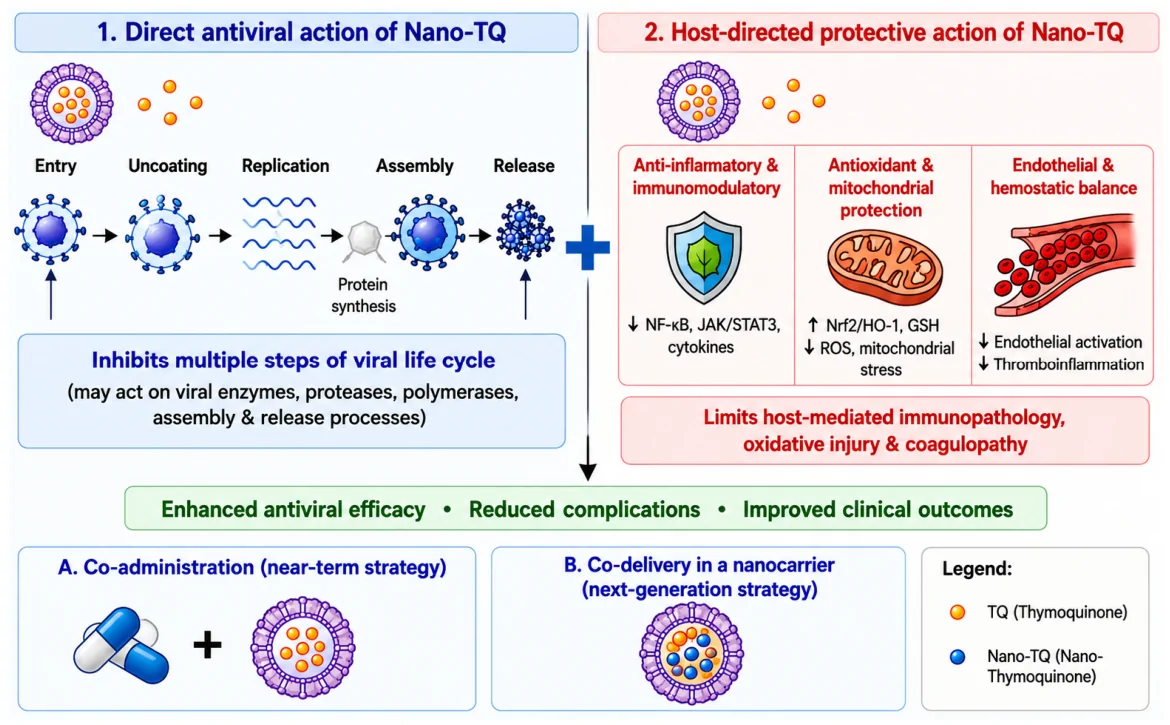
Conceptual representation of dual therapeutic role of Nano-TQ in viral infections. Nano-TQ may have potential virus-directed effects by influencing viral life cycle, while TQ-mediated modulation of inflammatory signaling and endothelial activation may help in limiting host-mediated injury. Together, these complementary actions could provide a basis for combining viral control with protection against virus-associated complications. Co-administration of Nano-TQ with established antiviral drugs may represent a promising near-term approach, whereas nanocarrier-based co-delivery is presented as a future concept. Importantly, these combination strategies, particularly nanocarrier-based co-delivery, have not yet been established for Nano-TQ in antiviral in vivo models.

**Figure 3:**
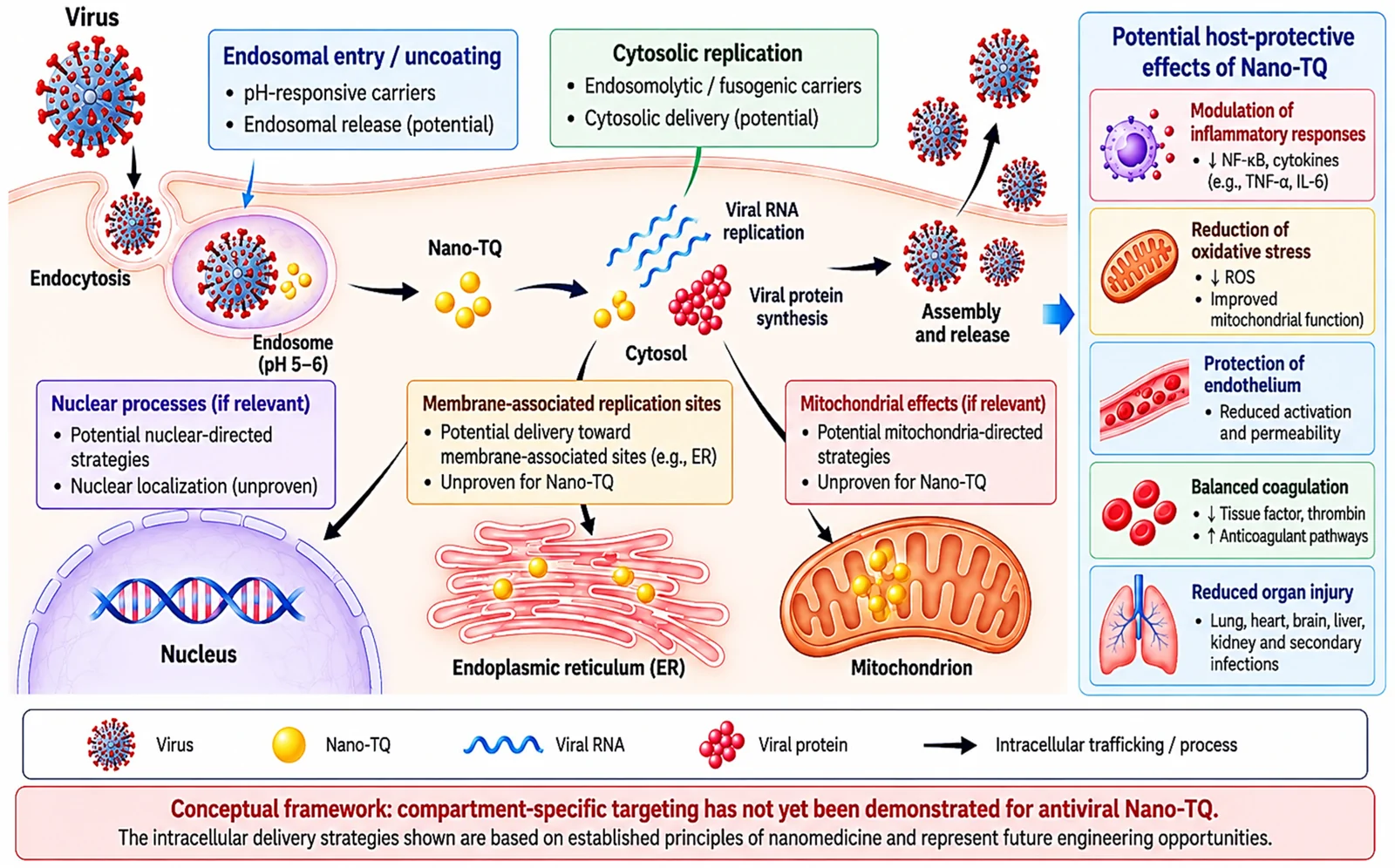
Proposed intracellular delivery opportunities for Nano-TQ based on general nanomedicine principles. Viruses use different intracellular environments during entry and replication, creating potential opportunities for virus-informed nanocarrier design. Endosomal, cytosolic, membrane-associated, nuclear, and mitochondrial delivery strategies shown here represent future engineering possibilities and have not been experimentally demonstrated for antiviral Nano-TQ. Their relevance should be established by linking intracellular TQ localization with target engagement and quantitative antiviral outcomes.

**Figure 4:**
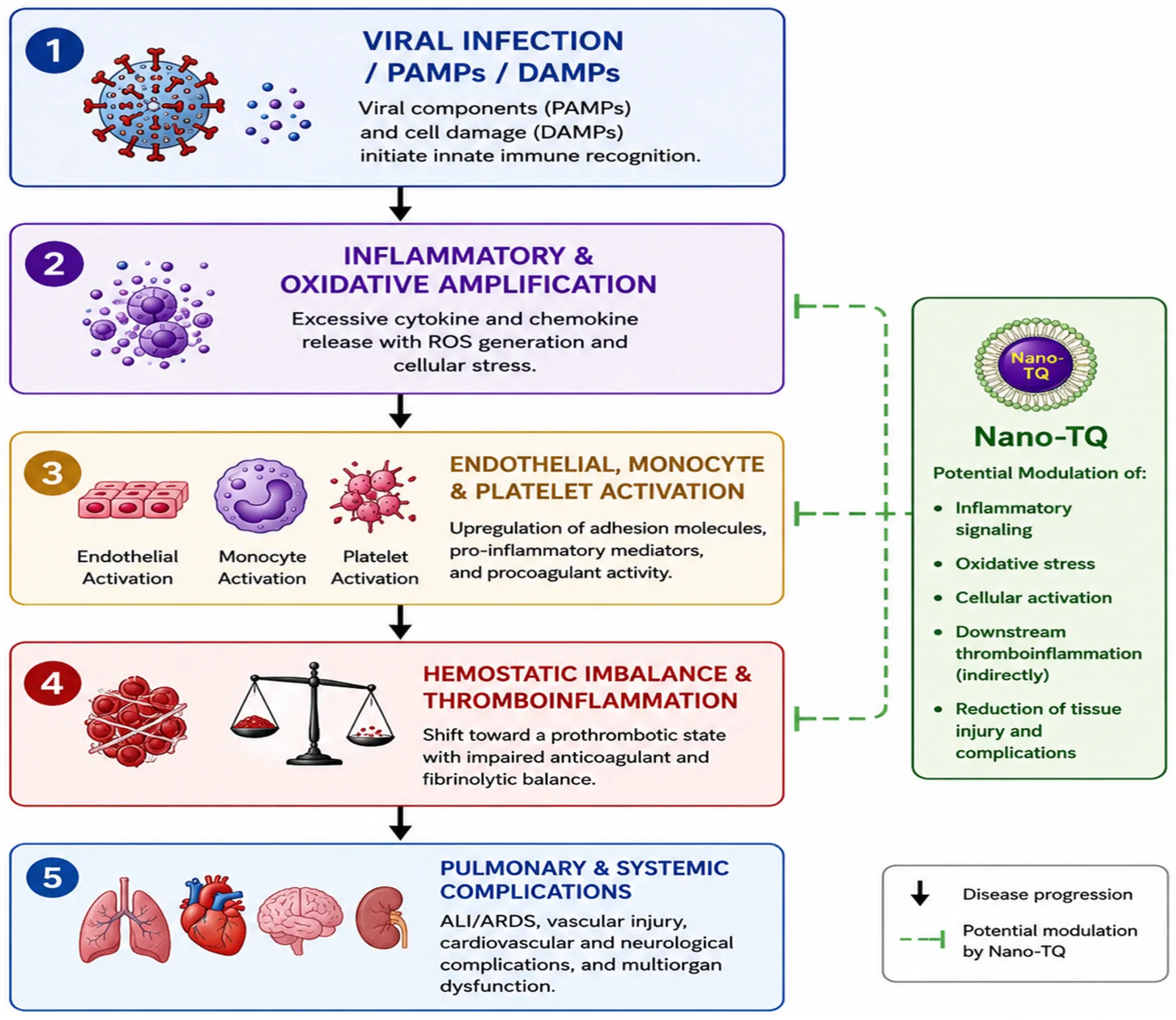
Proposed progression from viral infection to thromboinflammation and systemic complications and potential modulation by Nano-TQ. Viral components (PAMPs) and cell damage-associated signals (DAMPs) may promote inflammatory and oxidative amplification, followed by activation of endothelial cells, monocytes, and platelets. These responses can contribute to hemostatic imbalance and thromboinflammation, potentially leading to multiorgan complications. Nano-TQ may modulate upstream inflammatory, oxidative, and cellular responses and could thereby indirectly limit downstream thromboinflammation and tissue injury. Dashed lines indicate proposed Nano-TQ effects that require further experimental validation.

**Figure 5:**
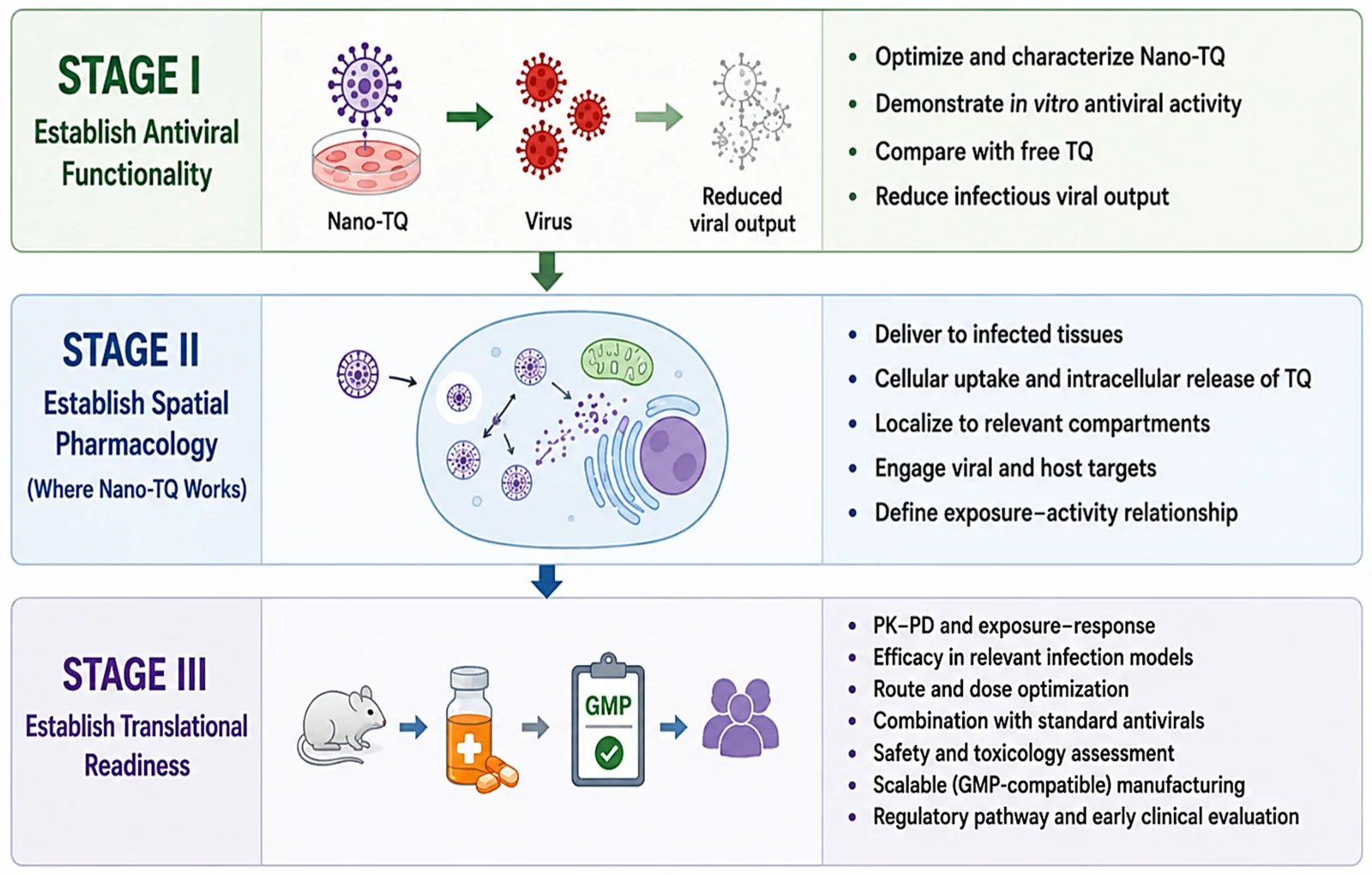
Translational roadmap for developing Nano-TQ as an antiviral nanomedicine. Proposed stepwise framework for translating Nano-TQ from experimental development toward potential clinical application. Stage I focuses on establishing antiviral activity relative to free TQ. Stage II emphasizes spatial pharmacology by linking delivery to infected tissues, intracellular TQ release, and viral or host target engagement with antiviral activity. Stage III addresses PK–PD relationships, efficacy in relevant infection models, route and dose optimization, combination therapy, safety, scalable manufacturing, regulatory requirements, and early clinical evaluation. Advancement between stages should ideally depend on reproducible therapeutic benefit at clinically achievable exposures.

**Table 1:** Current Evidence and Translational Rationale for Thymoquinone and Nano-Thymoquinone in Viral Diseases.

Viral System	Intervention	Evidence Type	Main Observation	TQ/Nano-TQ Concept	Major Limitation
Chikungunya virus [21]	Purified TQ	Experimental, cell-based and target-binding study	TQ interacted with the viral capsid protein and inhibited CHIKV replication; KD ≈ 27 μM and EC50 ≈ 4.48 μM	Provides one of the clearest examples of direct TQ-specific antiviral activity and supports a virus-directed mechanism	Evidence is virus-specific and does not establish broad activity against other alphaviruses
Coronaviruses [23,24]	TQ	Experimental and computational studies	TQ has been proposed to interfere with viral entry and virus-associated molecular targets	Supports investigation of TQ against coronavirus infection and potential early-stage viral processes	Mechanistic evidence remains incomplete and several findings are computational
SARS-CoV-2 [25,26]	TQ	Primarily computational/mechanistic predictions	Interactions with proteins involved in viral entry or replication have been proposed	Provides hypotheses for potential TQ–virus interactions	Requires confirmation using authentic viral infection models
Murine cytomegalovirus [22]	*N. sativa* oil	In vivo experimental study	Reduced viral burden in liver and spleen with enhanced antiviral immune responses	Supports the broader antiviral potential of *N. sativa*-derived bioactives	Effects cannot be attributed specifically to TQ
Hepatitis C virus [27,28]	*N. sativa*-based preparations	Limited clinical studies	Reduction in viral load and improvement in liver-related parameters were reported	Suggests possible clinical antiviral relevance of *N. sativa* preparations	Not purified TQ; limited ability to establish TQ-specific efficacy
HIV [29,30]	*N. sativa*-based preparations	Case reports	Reduced viral load and improved CD4^+^ T-cell responses were described	Provides preliminary clinical observations suggesting antiviral potential	Case-report evidence is weak and intervention was not purified TQ
Respiratory viral disease	Nano-TQ	Proposed translational strategy	Nanoformulation may improve pulmonary or systemic TQ exposure and facilitate cellular delivery	Provides a rationale for inhalable or systemically delivered Nano-TQ	Direct antiviral superiority over free TQ has not yet been established
Intracellular viral replication niches	Nano-TQ	Mechanistic/translational hypothesis	Nano-TQ may enhance infected-cell uptake, intracellular release, and access to endosomal, cytosolic, membrane-associated, or nuclear virus–host processes	Central basis of the proposed spatial-pharmacology concept	Selective infected-cell or subcellular targeting remains largely unproven
Virus-associated inflammation and oxidative injury [11,13]	TQ/Nano-TQ	Experimental pharmacological evidence, largely outside direct viral models	TQ modulates NF-κB, IRAK1, STAT3, Nrf2/HO-1, ROS, and endogenous antioxidant defenses	Supports host-protective potential during viral disease	Contribution of these mechanisms to antiviral efficacy requires virus-specific validation

Direct TQ-specific antiviral evidence appears strongest in selected models such as CHIKV, while broader support comes from *N. sativa* preparations, computational studies, and host-directed pharmacology. The potential antiviral advantages of Nano-TQ remain to be more clearly established through direct experimental studies.

**Table 2:** Evidence Strength for TQ and Nano-TQ in Antiviral Applications.

Evidence Area	Current Evidence	Evidence Level	Interpretation
Antiviral activity of TQ [21,24]	Direct antiviral activity demonstrated in selected viral models, including CHIKV	Established	Supports TQ-specific antiviral potential, but evidence remains virus-dependent
Modulation of inflammatory pathways by TQ [11,12,13,14]	Demonstrated across multiple experimental models	Established	Strong pharmacological evidence, although virus-specific contribution varies
Improved TQ solubility/stability/formulation by nanoencapsulation [15,17,35]	Demonstrated with liposomal, lipid, polymeric and related formulations	Established	Pharmaceutical advantage of Nano-TQ
Altered pharmacokinetics and biodistribution [15,17]	Demonstrated mainly in non-viral animal models	Established	Supports improved delivery but does not establish antiviral superiority
Improved antiviral efficacy of Nano-TQ over free TQ [10]	Direct comparative viral evidence remains limited	Emerging	Requires free TQ versus Nano-TQ comparison using quantitative virological endpoints
Delivery of Nano-TQ to infected cells [46,47]	Limited direct antiviral evidence	Emerging	Cellular delivery requires validation in virus-specific models
Functional intracellular delivery [47]	Not adequately demonstrated for antiviral Nano-TQ	Hypothetical	Uptake alone does not establish intracellular target engagement
Endosomal escape/subcellular targeting [47]	Proposed from nanocarrier principles	Hypothetical	Must be matched experimentally to viral replication biology
Virus-informed nanocarrier design [46,49]	Conceptual framework	Hypothetical	Requires virus-specific formulation and trafficking studies
Inhalable Nano-TQ for respiratory viruses [44,45,46]	Pharmacologically plausible but underexplored	Hypothetical/ Emerging	Requires pulmonary PK, antiviral efficacy and safety studies
Nano-TQ modulation of virus-associated complications [11,13,14]	Supported indirectly by TQ pharmacology	Emerging	Direct evidence in viral complication models remains limited
Nano-TQ plus approved antivirals [10,46]	Proposed adjunctive strategy	Hypothetical	Requires combination efficacy, interaction and safety studies

**Table 4:** Comparison of Nano-TQ with Approved Antiviral Agents.

Parameter	Approved Direct-Acting Antivirals	Nano-TQ
Antiviral potency	Generally, well defined for the indicated virus	Limited and virus-dependent; insufficient Nano-TQ data
Therapeutic index	Established during preclinical/clinical development	Not adequately established for antiviral Nano-TQ
Mechanism	Usually defined virus-specific target(s)	Potential virus-directed plus host-protective actions
Clinical evidence	Established for approved indications	No established antiviral clinical efficacy
Intracellular delivery	Drug- and virus-dependent	Potentially engineerable, but functional targeting remains unproven
Host-complication modulation	Usually not the primary therapeutic function	Potential complementary advantage, requiring validation
Accessibility	Varies among drugs and healthcare systems	Currently uncertain
Cost	Known or estimable for marketed products	Unknown; nanomanufacturing may offset low raw-material cost
Current therapeutic position	Standard/approved antiviral therapy	Experimental; potential future adjunct
Key evidence needed	—	Head-to-head potency, therapeutic index, PK–PD, safety, efficacy, and cost-effectiveness

## Data Availability

No new data were created or analyzed in this study. Data sharing is not applicable to this article.
